# A Compensatory Mutation Provides Resistance to Disparate HIV Fusion Inhibitor Peptides and Enhances Membrane Fusion

**DOI:** 10.1371/journal.pone.0055478

**Published:** 2013-02-05

**Authors:** Matthew P. Wood, Amy L. Cole, Piotr Ruchala, Alan J. Waring, Lisa C. Rohan, Preston Marx, Patrick M. Tarwater, Phalguni Gupta, Alexander M. Cole

**Affiliations:** 1 Department of Molecular Biology and Microbiology, Burnett School of Biomedical Sciences, University of Central Florida College of Medicine, Orlando, Florida, United States of America; 2 Department of Medicine, David Geffen School of Medicine, University of California Los Angeles, Los Angeles, California, United States of America; 3 Department of Infectious Diseases and Microbiology, School of Public Health, University of Pittsburgh, Pittsburgh, Pennsylvania, United States of America; 4 Magee Women’s Research Institute and Department of Pharmaceutical Sciences, School of Pharmacy, University of Pittsburgh, Pittsburgh, Pennsylvania, United States of America; 5 Tulane National Primate Research Center, Tulane University, Covington, Louisiana, United States of America; 6 Department of Biostatistics, Texas Tech University Health Sciences Center, El Paso, Texas, United States of America; University of Missouri, United States of America

## Abstract

Fusion inhibitors are a class of antiretroviral drugs used to prevent entry of HIV into host cells. Many of the fusion inhibitors being developed, including the drug enfuvirtide, are peptides designed to competitively inhibit the viral fusion protein gp41. With the emergence of drug resistance, there is an increased need for effective and unique alternatives within this class of antivirals. One such alternative is a class of cyclic, cationic, antimicrobial peptides known as θ-defensins, which are produced by many non-human primates and exhibit broad-spectrum antiviral and antibacterial activity. Currently, the θ-defensin analog RC-101 is being developed as a microbicide due to its specific antiviral activity, lack of toxicity to cells and tissues, and safety in animals. Understanding potential RC-101 resistance, and how resistance to other fusion inhibitors affects RC-101 susceptibility, is critical for future development. In previous studies, we identified a mutant, R5-tropic virus that had evolved partial resistance to RC-101 during *in vitro* selection. Here, we report that a secondary mutation in gp41 was found to restore replicative fitness, membrane fusion, and the rate of viral entry, which were compromised by an initial mutation providing partial RC-101 resistance. Interestingly, we show that RC-101 is effective against two enfuvirtide-resistant mutants, demonstrating the clinical importance of RC-101 as a unique fusion inhibitor. These findings both expand our understanding of HIV drug-resistance to diverse peptide fusion inhibitors and emphasize the significance of compensatory gp41 mutations.

## Introduction

Prevention of HIV transmission using safe and effective treatments with specific mechanisms of action remains a necessary challenge in the development of microbicides. Of the options currently being explored, HIV entry has become an attractive target for HIV treatment and prevention. Entry is a multi-step process in which interactions between viral and host proteins result in fusion of the enveloped virus with host membranes. Fusion of the host and viral membranes occurs through direct insertion of gp41 into the host membrane and subsequent formation of a trimer of gp41 hairpin complexes, composed of the heptad repeat regions 1 and 2 (HR1 and HR2). The formation of this stable complex, referred to as a 6-helix bundle, brings the viral and host membranes into close enough proximity for fusion to occur [1], [2].

During membrane fusion, conformational changes in the envelope proteins provide a kinetic window for inhibition by drugs that bind to the gp41 ectodomain [3], [4]. One such drug, enfuvirtide (ENF), is an anionic, 36-amino acid peptide that competes with the HR2 region of gp41 for binding to HR1, thus preventing formation of the mature gp41 6-helix bundle required for fusion [5]. Currently, enfuvirtide (ENF) is the only fusion inhibitor approved for HIV treatment, and resistant viruses continue to emerge [6], [7].

Another class of antiviral peptides that has been shown to act as fusion inhibitors are retrocyclins [3], [8], [9]. These are synthetic, 18-residue, cyclic antimicrobial peptides that possess amino acid compositions and structures based on the theoretical product of human θ-defensin pseudogenes. Retrocyclins have been found to inhibit HIV-1 infection in both *in vitro* and in *ex vivo* models and have been shown to exhibit antiviral activity against both R5 and X4 tropic clinical isolates of HIV-1 [10], [11]. Retrocyclins have also retained their antiviral activity for over 1 week following application in non-human primates [12]. Further, retrocyclins remain stable under acidic conditions, are resistant to boiling, and lack cytotoxic and proinflammatory activity at concentrations over 100 times their IC_50_ [13], [14]. Because of its unique stability and safety, combined with its potent anti-HIV activity even in the presence of mucosal fluids, the retrocyclin analog RC-101 is currently being developed as an intravaginal microbicide to prevent sexually transmitted HIV-1.

Retrocyclins prevent viral membrane fusion by binding the HR2 helix of gp41 [3], [8]. Using multi-round, serial passaging of the HIV-1 R5 strain, BaL, in the presence of sub-inhibitory concentrations of the RC-101, we selected for partially-resistant mutants. In agreement with retrocyclins preventing gp41 activity, mutations in gp41 alone were shown to be sufficient for RC-101 resistance in pseudotyped viruses. These mutations identified in gp41 were Q66R and N126K, located in the HR1 and HR2 regions, respectively [9]. Due to the cationic nature of these mutations, it was presumed that they might act to electrostatically repel the cationic RC-101 peptides.

Here, we sought to delineate the mechanism by which mutations in gp41 contribute to RC-101 resistance. Specifically, we determined that Q66R compromises gp41 fusion and entry kinetics, and that N126K behaves as a compensatory mutation to enhance gp41 activity in RC-101 resistance, as has been observed in resistance to ENF [15]. This is the first time that mutations compromising gp41 activity, followed by a compensatory mutation, have been observed as a pattern of drug resistance used to evade a non-gp41-mimetic peptide. Additionally, we identified the activity of RC-101 against clinically relevant enfuvirtide-resistant mutants.

## Materials and Methods

### BaL *env* Molecular Clones and DNA Constructs

The pNL43 plasmid encodes an infectious X4 strain of HIV-1 [15]. Briefly, pNL43 was digested using EcoR1 and XhoI prior to gel purification of the 11 kb cleavage product using the QIAEX II system (Qiagen, Valencia, CA). For the construction of infectious molecular clones containing the BaL *env* sequence, viral RNA was isolated from infected PM1 cell supernatants containing high titers of BaL as determined by gag p24 concentration using a p24 ELISA (Perkin-Elmer, Waltham, MA). Viral cDNA was generated from purified RNA using the Superscript III system (Life Technologies, Carlsbad, CA). *env-*containing regions were enriched using oligonucleotide primers (FWD 5′-CTGCAACAACTGCTGTTTATCC-3′ REV 5′-GATACTGCTCCTACTCCATCTGCT-3′) designed to target the 3′ region of *vpu* and the 5′ region of *nef*. Cloning inserts were then purified from PCR products using crystal violet gel purification with QIAquick columns (Qiagen). Purified DNA was then inserted into the TOPO-XL cloning vector and sequenced using the M13F and M13R priming sites as well as *env*-specific sequencing primers [9]. After sequencing, *env* inserts were prepared for InFusion cloning by adding EcoRI and XhoI –specific extensions in a second round of amplification (FWD 5′-GCCATAATAAGAATTCTGCAACAACTGCTGTTTATCC-3′ REV 5′-TTTTCTAGGTCTCGAGATACTGCTCCTACTCCATCTGCT-3′). The BaL *env*- containing PCR product was then cloned into the pNL43 plasmid backbone using the InFusion cloning system (Clontech, Mountain View, CA). This method was employed to avoid complications due to non-conserved restriction sites between pNL43 and BaL, thus preserving the complete BaL *env* sequence. The pMONO-neo GFP vector (Invivogen, San Diego, CA) was transfected into 293T cells and used for visualization of cell-cell fusion.

### Virus and Cell Culture

The following cell lines were obtained from the NIH AIDS Research and Reference Reagent Program, Division of AIDS, NIAID, NIH: TZM-bl from Dr. J.C. Kappes (University of Alabama, Birmingham, AL) and Dr. X. Wu (University of Alabama, Birmingham, AL and Tranzyme, Research Triangle, NC) and PM1 cells from Dr. M. Reitz (Institute of Human Virology, Baltimore, MD). 293T cells were purchased from the American Type Culture Collection (ATCC). TZM-bl reporter cells and 293T cells were maintained in DMEM supplemented with 10% fetal bovine serum with penicillin and streptomycin. PM1 cells were maintained in RPMI 1640 media supplemented with 20% fetal bovine serum with penicillin and streptomycin. Cells were stored at 37°C and 5% CO_2_. Virus from molecular clones was produced by transfecting 293T cells with viral DNA using the Effectene (Qiagen) following the manufacturer’s instructions. Viral 293T supernatants were collected 24 hours after transfection and immediately stored at −80°C.

### Antiviral Peptides and Synthesis

The 18 amino acid RC-101 peptide was synthesized as previously described [9], [16]. ENF was provided by the NIH AIDS Research and Reference Reagent Program (Trimeris/Roche) and was reconstituted in water prior to storage at −20°C. The α-defensin HNP-1 was purchased from Bachem (Torrance, CA) and was reconstituted in water prior to storage at −20°C. HIV inhibitory peptides Grifonin-1 and RC107GG-F2 were produced by solid-phase FMOC chemistry as previously described [17], [18].

### Antiviral Activity Assays

TZM-bl luciferase reporter cells were used to determine inhibition of infection as previously described [9]. TZM-bl cells were plated in 96-well, black, transparent bottomed plates at a density of 5×10^4^ cells per well in 100 µL of the previously described culture media. 24 hours after plating, media was removed and replaced with peptide or vehicle control in 50 µL culture media before incubation for 10 minutes at 37°C and 5% CO_2_. Viral stocks were titered using β-galactosidase expression in TZM-bl reporter cells infected with serial dilutions of wild-type and mutant HIV-1 molecular clones using a modified MAGI cell assay [19]. Briefly, TZML-bl cells were plated at 1.0×10^3^ cells/well in a 96-well dish prior to 24-hour incubation. Cells were then infected in quadruplicate with dilutions of viral stocks ranging from 4^−3^–4^−12^. After 48 hours of infection, cells were washed, fixed, and stained with Xgal for β-galactosidase activity. Infected blue cells were counted in each dilution and TCID50 values were then calculated using the method of Reed and Muench [20]. In experiments with antiviral compounds, virus was used at MOI 0.2 for each viral genotype tested. In experiments using HNP-1, Grifonin-1, and RC107GG-F2, serum-free conditions were used for the initial 4 hours of infection, before replacement of media with DMEM supplemented with 10% FBS, in order to observe antiviral activity. Infected cells were incubated for 24 hours before removal of media and lysis using Glo-Lysis buffer (Promega, Madison, WI). Plates containing lysed cells were sealed in parafilm and stored at −80°C for 1 to 24 hours before thawing and treating with 100 µL per well of Bright-Glo reagent (Promega). Bioluminescence was measured using an Lmax luminometer (Molecular Devices, Sunnyvale, CA). RLUs were normalized to baseline expression in uninfected cells.

### Viral Fitness Assays

PM1 infections were carried out in 100 µL of previously described culture media in deep 1ml volume 96-well plates. 1×10^5^ PM1 cells were incubated with peptide or vehicle control for 10 minutes before infection using an MOI of 0.003. Cells were then incubated at 37°C and 5% CO_2_ for 2 hours, agitating every 30 minutes. Conditions with virus and peptide or with vehicle control were diluted with the addition of 900 µL culture media to each well. Treated cells were then centrifuged 200×g for 5 minutes. Media containing virus and peptide or control was aspirated and cell pellets were re-suspended in 0.5 ml culture media containing peptide or control at experimental concentrations. Media treated with drugs or vehicle controls was changed on days 3 and 5 with the volume of cultures being increased to 1 ml on day 3. Supernatants were collected on day 7 and stored at −80°C. Levels of p24 in samples were determined using an HIV-1 p24 ELISA.

### Cell-Cell Fusion Assay

Fusion efficiency of wild type and mutant envelope proteins was determined using a cell-cell fusion assay consisting of Env- and GFP-expressing 293T effecter cells and luciferase-expressing TZM-bl reporter cells. 293T cells were transfected with equivalent molar concentrations of both molecular clones and GFP or GFP alone in 24-well dishes. The protease inhibitor saquinavir was used at 1 µM concentrations to prevent virion maturation while allowing envelope expression and processing in effector cells. Twenty-four hours after transfection, 293T cells were washed, trypsinized, and co-cultured with TZM-bl cells using a 1∶1 ratio of 2×10^4^ cells each in black 96-well plates. After 6 hours of co-culture, cells were lysed and stored at −80°C overnight. Luminescence was measured using an Lmax luminometer (Molecular Devices). Alternately, transfected cells were co-cultured with TZM-bl cells grown on 8 well chamber slides (Lab-Tek, Bloomington, IN) for 6 hours before fixing with 4% paraformaldehyde and washing twice with PBS. Coverslips were mounted on fixed cells using DAPI-containing mounting solution (Vector Laboratories, Burlingame, CA). Images were acquired using a Zeiss Axiovert inverted fluorescent microscope with Axiovision software.

### Determination of Entry Kinetics

TZM-bl reporter cells were plated in black 96-well plates at 2.5×10^4^ cells per well. After 24 hours, cell supernatants were removed and replaced with 50 µl fresh media. Suspensions of equivalent concentrations of tested virus were quickly added to each well before centrifugation of plates at 2095×g for 30 minutes at 4°C as previously described [21]. Plates were then kept at 37°C for 0–1 hour, and infection was stopped at individual time points by adjusting the concentration of ENF in experimental wells to 5 µg/ml. This concentration was found to not affect cell metabolism via MTT assay. Plates were incubated for 24 hours before lysing cells and measuring bioluminescence as described above.

### Sequence and Structural Analysis

Mutations in gp41 were observed and confirmed using eBioX Software for Mac OSX to generate alignments and concatenate Sanger Sequencing data [22]. Structural analyses of gp41 mutations were carried out using Mac PyMOL (DeLano Scientific).

### Statistical Analysis

All analyses were performed using GraphPad Prism v4.0c. TZM-bl antiviral assays were analyzed using an unpaired student’s t-test or two-way ANOVA with bonferroni correction. Viral fitness assays and differences in cell-cell fusion were analyzed using a paired student’s t-test to account for inter-assay variability. Differences in entry kinetics were determined using linear regression to compare slopes.

## Results

### RC-101 Selects for Enfuvirtide-resistant HIV-1

**Figure 1:**
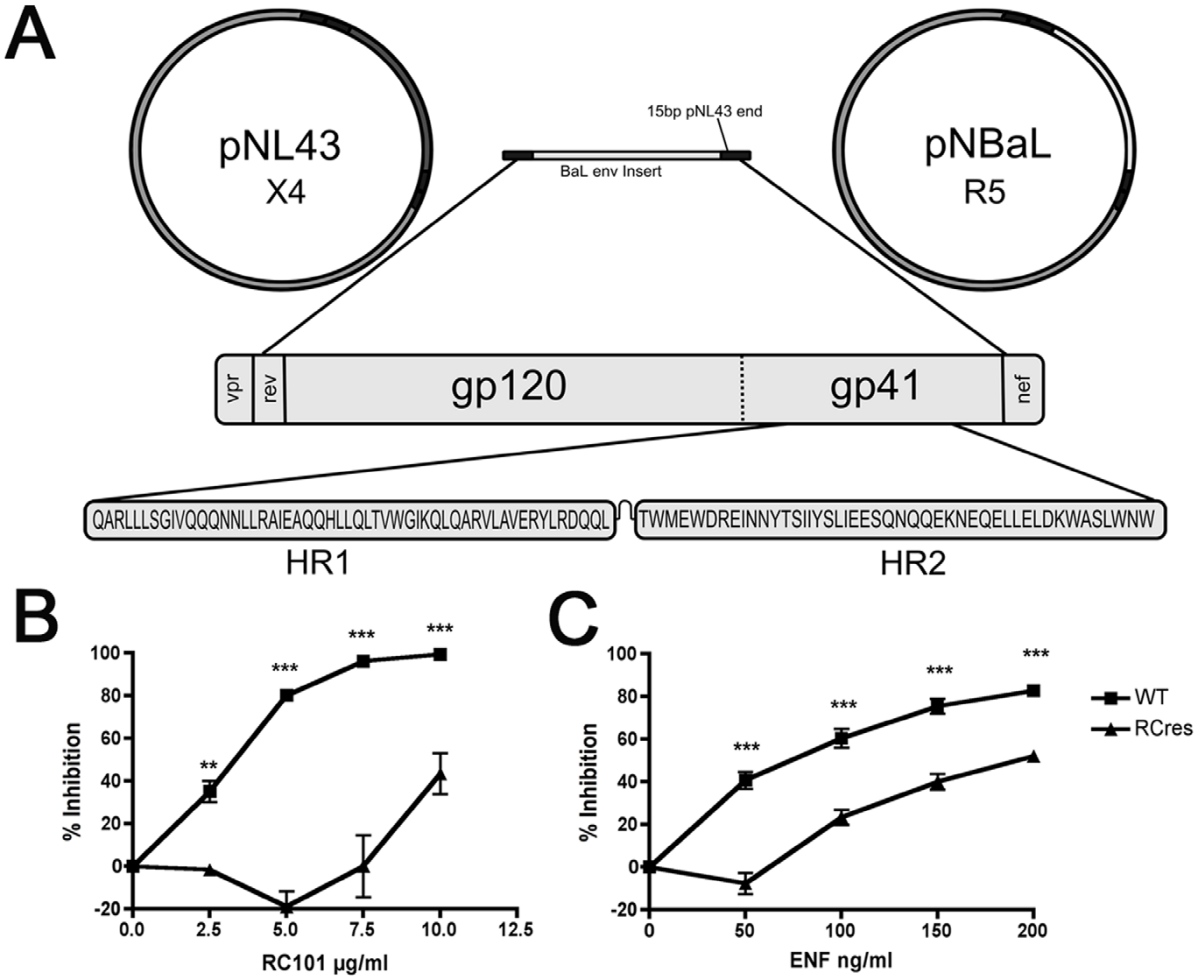
Synthesis and Characterization of BaL *env* Molecular Clones. To study the effect of gp41 mutations identified in the BaL envelope, the dominant *env* genotypes from untreated and RC-101-passaged virus were used to generate the pNBaL molecular clone containing the complete BaL envelope within pNL43 (**A**). Molecular clones possessing the envelope proteins cloned from untreated BaL (WT) or from RC-101-passaged BaL (RCres) were treated with either RC-101 (**B**) or ENF (**C**). Error bars represent SEM. Differences in percent inhibition were determined between WT and RCres at each drug concentration (N = 3; ** = *p*<0.01, *** = *p*<0.001).

Infectious molecular clones containing the complete BaL R5 envelope gene were produced using InFusion cloning. This method utilizes 15 base pair single-stranded DNA present at the 5′ ends of the insert and vector DNA, which allows cloning of PCR products into plasmids based on areas of homology (**Figure 1A**). This allowed cloning of BaL *env* into the pNL43 vector despite differences in restriction enzyme recognition sites. Chimeric BaL-pNL43 HIV-1 constructs (pNBaL) were generated containing both the wild type and RC-101-passaged *env* genes. The molecular clone produced from the RC-101-passaged *env* (referred to as RCres) contained the gp41 mutations Q66R and N126K, in addition to several mutations in the variable regions of gp120.

Equivalent amounts of virus, as determined using p24 proteins levels, were used to infect TZM-bl cells in the presence or absence of increasing concentrations of both RC-101 and ENF or a vehicle control (**Figure 1B and 1C**). Wild type pNBaL was almost completely inhibited by RC-101 at 10 µg/ml and by ENF at 200 ng/ml. Mutant pNBaL containing multiple *env* mutations showed significant resistance to both drugs at varying concentrations and was inhibited by approximately 50% by both RC-101 at 10 µg/ml and ENF at 200 ng/ml.

While RC-101 and ENF are structurally and chemically disparate drugs, these results demonstrate that a mutant virus selected by RC-101 also displays partial resistance to ENF. This could be explained by the presence of the N126K mutation in the HR2 region of the RC-101-resistant virus. Previously, this amino acid mutation in HR2 has been found to occur with the ENF mutation V38A in HR1, where it has been attributed to an increased rate of fusion and a potential reduction in the kinetic window in which ENF, and presumably other gp41-mimetic peptides, can exert their activity [23], [24]. Individual mutations within the RC-101-resistant gp41 would have to be explored further in order to explain the observed cross-resistance.

### Resistance Mutations in HR1 are Drug-specific While an HR2 Mutation Provides Variable Cross-resistance

To determine which specific mutations contributed to drug resistance, we introduced the two gp41 mutations observed in RC-passaged BaL, Q66R and N126K, into the wild-type BaL *env*. We also generated mutants containing the well-characterized ENF resistance genotypes V38A and V38A+N126K. These mutants were used to infect TZM-bl reporter cells at MOI 0.2, in the presence and absence of either RC-101 or ENF at varying concentrations.

**Figure 2:**
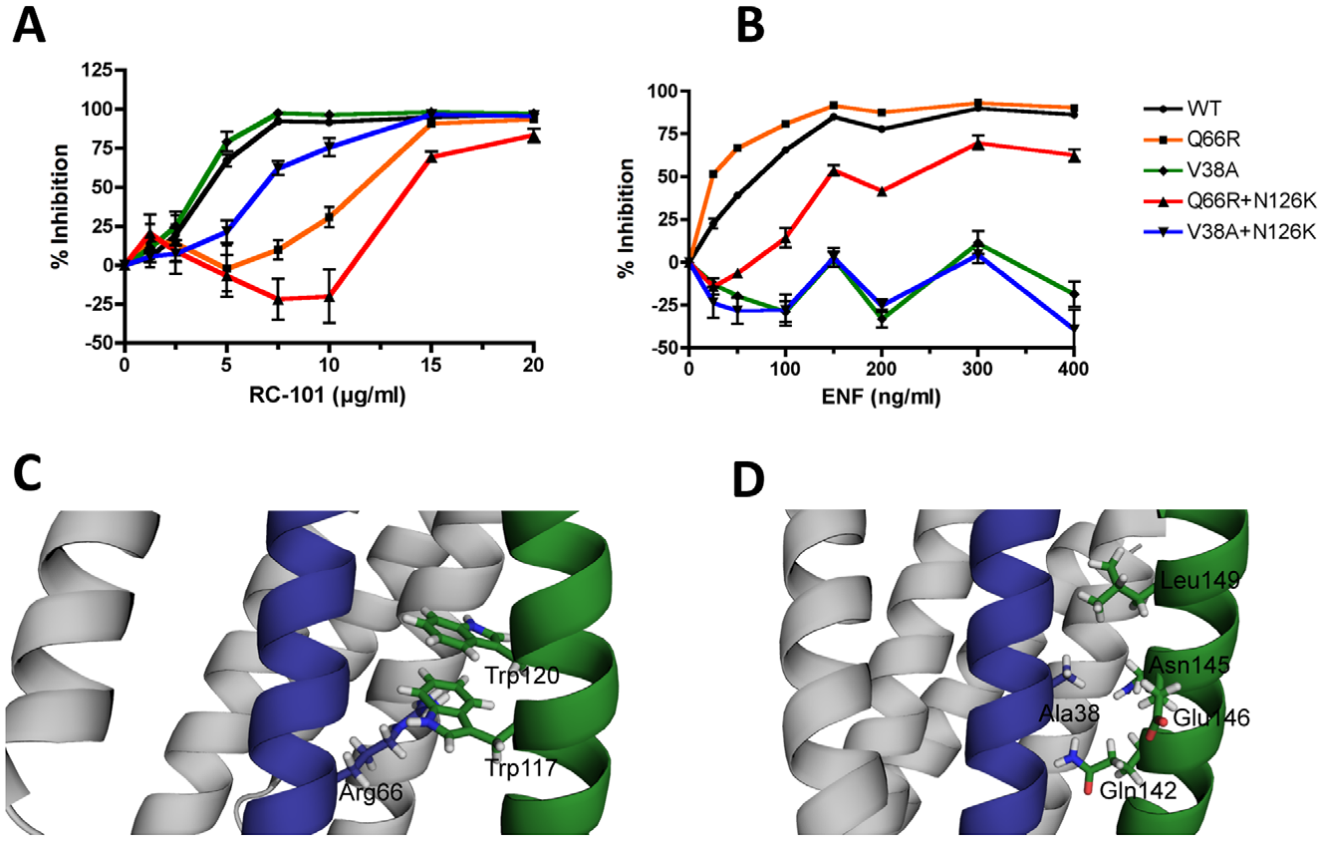
Effect of HR1 and HR2 Mutations on Drug-Resistance. The role of individual gp41 mutations on drug-resistance was determined using site-directed mutagenesis of the pNBaL vector. The gp41 mutations identified in RC-101 resistant virus, Q66R and Q66R+N126K, were used to understand contributions to drug resistance. We also tested mutations shown to confer resistance to ENF, V38A and V38A+N126K. Equal concentrations of infectious virus, as determined by TCID50, were used to inoculate TZM-bl cells with increasing concentrations of RC-101 and ENF. Error bars represent SEM. Percent inhibition was calculated from RLU values and normalized to vehicle control (**A**) (N = 3−5). The structure PDB 1IF3 was used to model the positions of primary gp41 mutations. HR1 (blue) mutations Q66R (**B**) and V38A (**C**) are shown along with nearby residues on HR2 (green) of the same gp41 molecule. Two additional gp41 molecules making up the 6-helix bundle are shown in gray.

As expected, the wild-type virus exhibited susceptibility to both RC-101 and ENF (**Figure 2A and 2B**). The Q66R mutant demonstrated resistance to RC-101, with an IC50 2.3-fold higher than the wild-type virus. At the same time, the IC50 of ENF against Q66R was 2.3-fold lower than the wild-type virus. The Q66R+N126K double mutant possessed greater resistance to RC-101 than the wild-type virus (3.1-fold) and than Q66R alone (1.4-fold). Q66R+N126K also demonstrated increased infection at 10 µg/ml versus control conditions, but could be inhibited >80% at 20 µg/ml. These observations suggest that the addition of N126K improves resistance to RC-101.

Conversely, the V38A mutant was resistant to the highest concentrations of ENF tested, yet remained susceptible to RC-101. The V38A+N126K double mutant was also resistant to ENF at all concentrations tested, and additionally demonstrated 1.6-fold greater resistance to RC-101 than the wild-type virus. The HR1 mutations Q66R and V38A were analyzed using the theoretical structure of the mature gp41 protein and both were found to be present on the same region of the HR1 helix responsible for the intramolecular association with HR2 (**Figure 2C and 2D**) [**25**]. While both mutations appeared at the same region of the HR2 helix, V38A and Q66R are located near the N and C termini of HR1, respectively, potentially contributing to the specificity for either drug tested.

### The HR2 Compensatory Mutation N126K Provides Increased Fitness in the Presence of Fusion Inhibitors

To determine the effect of HR1 and HR2 mutations on viral replication, we subjected our mutants to 7-day viral fitness assays using lymphoblast-derived PM1 cells. Cells were infected in the presence of drugs or vehicle control using a relatively low titer (MOI 0.003). This ensured that the majority of infection would occur over a seven-day period, allowing us to determine the effects of each mutant on both drug resistance and on the overall replication fitness in culture.

**Figure 3:**
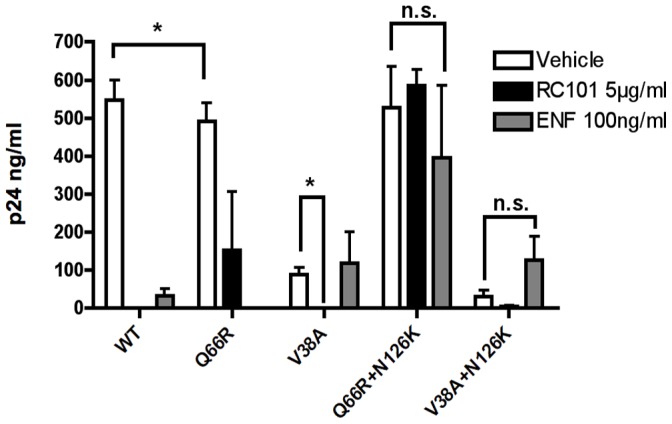
Analysis of Viral Fitness and Drug Resistance. To determine if N126K is acting as a compensatory mutation in RC-101, viral fitness in culture over a 7 day period was determined for wild-type (WT) and drug-resistant mutants in the presence or absence of RC-101 and ENF. An MOI of 0.003, as determined by TCID50, was used for the initial inoculation of PM1 cells. Fitness was determined by p24 concentration measured in cell supernatants. Error bars represent SEM. Black lines indicate comparisons between conditions (N = 3; * = *p*<0.05).

In vehicle control conditions, virus containing the wild-type *env* consistently infected to a greater degree than the HR1-only mutants Q66R or V38A (**Figure 3**). In agreement with TZM-bl results, Q66R remained partially resistant to RC-101 at 5 µg/ml, and completely susceptible to ENF. Oppositely, the V38A mutant was significantly inhibited by RC-101, yet remained resistant to ENF.

When the HR2 mutation N126K was combined with Q66R, this double mutant no longer demonstrated significant replication differences from wild type virus in vehicle treated conditions, suggesting restored fitness. Furthermore, N126K imparted increased RC-101 and ENF resistance to the Q66R mutant. However, for the V38A mutant virus, N126K did not restore viral fitness in vehicle control conditions; instead, this double mutant suffered slightly reduced fitness compared to the V38A mutant virus (p<0.05). The secondary N126K mutation appeared to provide an increased ability to infect in the presence of ENF, though this trend was not statistically significant.

The effects of the N126K mutation in these experiments suggest that it acts as a compensatory mutation to restore viral fusion to the RC-101-resistant mutant Q66R. This observation suggests that N126K could be acting to increase the rate of gp41 fusion, potentially leading to a previously characterized hyperfusogenic phenotype observed during ENF resistance, but not understood to be implicated in RC-101 resistance [23].

### N126K Differentially Enhances gp41 Fusion Efficiency Compromised by Drug Specific HR1 Mutations

Our previous results suggest that the N126K mutation provides resistance to both fusion inhibitors tested. Based on this, we speculated that an increase in gp41 fusion efficiency could both restore fitness lost in the presence of inhibitors and provide resistance by fusing more rapidly, thus decreasing the possible time of interaction with fusion inhibitors. To observe potential differences in gp41 fusion between mutants, cell-cell fusion assays were performed.

**Figure 4:**
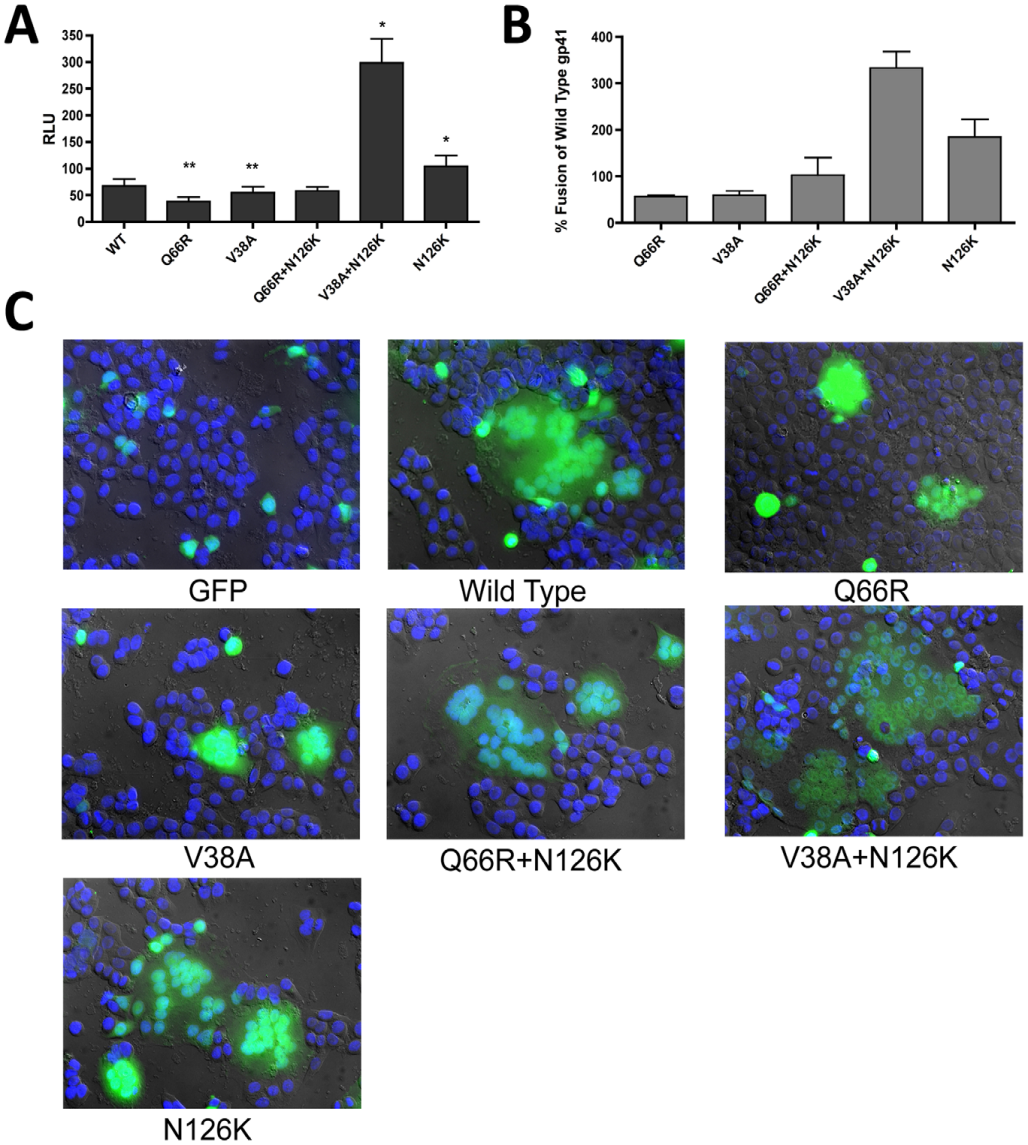
Effect of HR1 and HR2 Mutations on Cell-Cell Fusion. Cell-cell fusion was analyzed to determine differences in gp41 fusion demonstrated by wild type (WT) and drug-resistant mutant virus. Transformed 293T cells expressing BaL Env and Tat proteins, as well as GFP, were co-cultured with TZM-bl reporter cells. RLU values correlate with cell-cell fusion during co-culture (**A**), which is also shown as percent fusion of WT (**B**). Error bars represent SEM. Fusion for each mutant virus was compared to that observed in WT (N = 4; or 5 * = *p*<0.05, ** = *p*<0.01). Fluorescent imaging and DIC were used to observe syncytia formation as a secondary indicator to compare cell-cell fusion between mutants (**C**).

Cell-cell fusion was quantified by luciferase expression using target reporter TZM-bl cells following fusion with HIV Env- and Tat-expressing effector 293T cells. Cell-cell fusion was found to decrease by roughly half for both the Q66R and V38A HR1 mutants when compared with wild-type *env* (**Figure 4A and 4B**). Cells expressing N126K alone demonstrated a higher degree of fusion when compared with the wild-type *env*, while the Q66R+N126K combination restored fusion to roughly that of wild-type *env*. Interestingly, V38A+N126K displayed a 3.5-fold increase in fusion from wild-type, well beyond any of the other genotypes tested. In addition to data obtained by luciferase readings, imaging of GFP and *env* expressing cells revealed multinucleated fusion products whose sizes were proportional to cell-cell fusion as determined by luciferase readings and provided visual evidence comparing fusion between gp41 mutants (**Figure 4C**). Large syncytia were consistently more frequent in N126K- and V38A+N126K-expressing cells than with other genotypes. Conversely, effector cells expressing Q66R and V38A displayed numerous small, bright syncytia formed by relatively few cells. Together, these results reveal that the N126K mutation compensates for a loss of fusion due to either the Q66R or V38A primary mutations.

### N126K Restores Viral Entry Kinetics Compromised by Q66R

**Figure 5:**
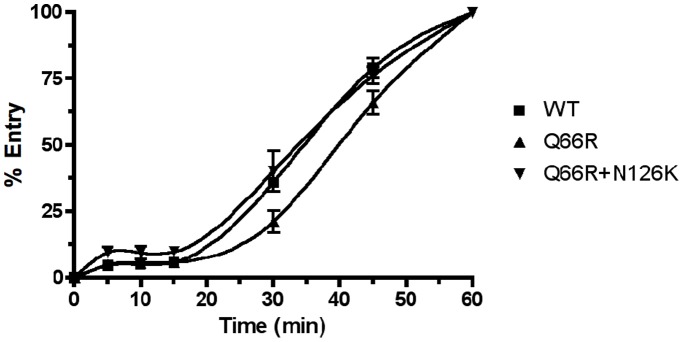
Entry Kinetics of RC-101-Resistant Virus. Viral entry was observed over one hour to determine differences in entry kinetics between wild type (WT) and RC-101-resistant virus. Equal concentrations of infectious virus were used to infect reporter cells and infection was halted at specific time-points. Entry kinetics is shown as percent of total entry and graphed using cubic splines. Linear regression curves were fit to data and slopes were compared between the 15 and 30-minute time intervals. Error bars represent SEM. Both wild type (WT) and Q66R+N126K had significantly greater slopes than the Q66R mutant (N = 4; *p*<0.05).

While our results demonstrate an increase in membrane fusion associated with the N126K mutation, we can only speculate as to how this may affect entry and possibly drug resistance. Previous studies suggest that a potential increase in the rate of gp41 fusion and entry is associated with drug resistance and can decrease the window of time when fusion inhibitors can act on the prefusion complex [23]. To determine if our RC-101-resistant mutants displayed differences in entry, we next investigated whether N126K could modify the rate at which our R5 molecular clones infected cells. Entry kinetics assays were carried out using equal amounts of infectious viral clones representing WT, Q66R, or Q66R+N126K *env* genotypes **(** **Figure 5** **)**. There was a consistently sharp increase in infection by the WT and Q66R+N126K viruses at 30 minutes after transferring the cells from 4°C to 37°C to initiate infection. The Q66R single mutant virus demonstrated a significant lag in infection compared to both the WT and Q66R+N126K mutants at the points between 15 and 30 minutes. This experiment demonstrates that Q66R compromises the early stages of entry, and that N126K restores viral entry kinetics to levels observed in the WT.

### N126K Provides Resistance to other Structurally Diverse Peptide Entry Inhibitors

**Figure 6:**
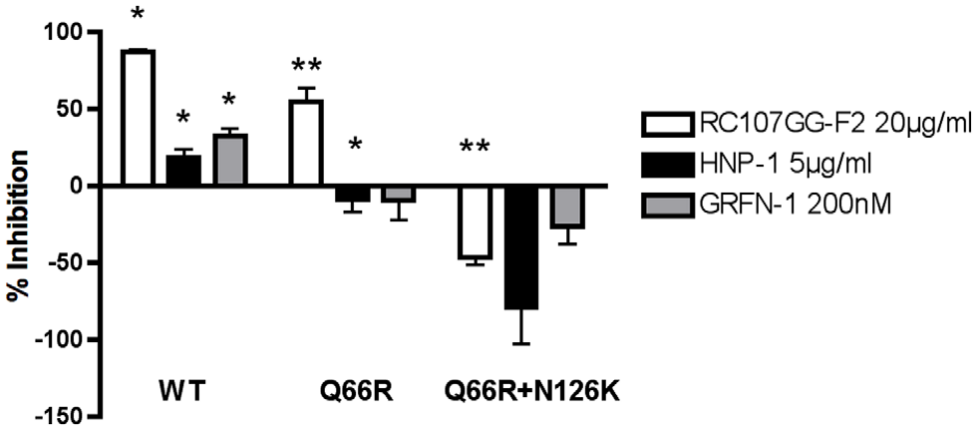
Resistance to Alternative Peptide Entry Inhibitors in RC-101-Resistant Virus. Susceptibility to antiviral peptides in wild type (WT) and RC-101-resistant mutants was determined using infection TZM-bl reporter cells. The antiviral peptides RC107GG-F2, HNP-1, and Grifonin-1 (GRFN-1) were used at concentrations known to inhibit infection by wild type virus. Percent inhibition of infection was calculated from RLU values and normalized to vehicle controls. Error bars represent SEM. Percent inhibition values were compared with infection using vehicle control alone (N = 3; * = *p*<0.05, ** = *p*<0.01).

Our previous experiments indicate that the N126K mutation acts to restore fusion and rate of entry while providing RC-101 and ENF resistance. Since both RC-101 and ENF are thought to use different mechanisms for fusion inhibition, we hypothesized that this mutation would also provide resistance to other unique peptide entry inhibitors. Using the previously described TZM-bl viral inhibition assay, we infected cells with either wild-type, Q66R, or Q66R+N126K *env* mutants in the presence of RC107GG-F2, Grifonin-1, or the human α-defensin HNP-1 at their reported EC50 concentrations. These peptides have all been identified as HIV-1 entry inhibitors and are believed to work through diverse mechanisms targeting the viral envelope [18], [26], [27]. Both Q66R and Q66R+N126K viruses displayed varying resistance to the antiviral peptides tested when compared to the wild-type virus (**Figure 6**). Alone, Q66R was able to provide resistance to both HNP-1 and Grifonin-1 at concentrations sufficient for inhibition of the wild-type virus, yet it remained susceptible to inhibition by RC107GG-F2. With the addition of N126K, we observed not only improved resistance to all peptides tested, but we saw increased infection in the presence of these peptides. These results demonstrate that the compensatory mutation N126K can provide improved resistance to a diverse set of peptides that act through inhibition of viral entry.

## Discussion

We observed that while single HR1 mutations provide some degree of drug resistance, this resistance is specific for either RC-101 or ENF. The V38A substitution, in particular, has been shown to be associated with resistance against multiple C-peptide type fusion inhibitors, yet we found V38A mutants remained susceptible to inhibition by RC-101. This is likely due to different gp41-binding regions for the two drugs, as ENF aligns with its corresponding HR1 residues closer to the N-terminus of gp41 and RC-101 is known to bind to HR2 [28]. The V38A single mutant remaining susceptible to RC-101 provides evidence that RC-101 targets a region outside of the ENF binding site. This finding is particularly promising since RC-101, and similarly acting θ-defensins, would likely remain active against HIV-1 harboring other ENF-resistance mutations such as those frequently observed in the “GIV” region of HR1 [29], [30].

Irrespective of the specificity of either HR1 mutation, N126K provided some degree of resistance to both drugs despite the clear differences in not only the structure of both inhibitors tested, but also the different binding sites of these peptides on gp41. Moreover, we have shown that N126K has a different effect on fusion depending on the primary mutation present in HR1 responsible for providing drug resistance. The effect on fusion associated with Q66R and V38A may again be explained by their similar locations on the HR1 helix. Both Q66R and V38A occupy the same position of the helical turn directly interacting with the HR2 helix of the same molecule and could reasonably affect membrane fusion in a similar way. The comparable effect on fusion seen with HR1 mutations is contrasted by the difference in compensatory activity with the addition of N126K. While the V38A+N126K genotype appears to overcompensate for the loss of fusion associated with V38A, Q66R+N126K restores fusion to the level of the wild-type virus. This observation may explain differences in fitness in the absence of fusion inhibitors displayed by the two double-mutants. Previous studies have demonstrated that N126K leads to a rapidly fusing gp41 that is dependent on ENF for infection. However, with our Q66R+N126K mutant we observed that fitness was not affected and that fusion and entry kinetics were restored to the levels of the wild-type virus. This observation is perhaps due to the differences in how Q66R or V38A would affect the formation of the mature gp41 complex. The difference being, that while V38A merely exchanges one small hydrophobic residue for another, perhaps reducing HR1’s affinity for both HR2 and ENF, Q66R inserts a large cationic residue into the hydrophobic pocket of gp41 that could not only act to repel a cationic peptide such as RC-101, but would also sterically constrain two key tryptophan residues located in the corresponding region of HR2 that are believed to play a significant role in the activity of gp41 [2]. This key difference may explain why we see overcompensation in fusion observed with V38A+N126K, while Q66R+N126K only exhibits restored fusion when compared with the wild type.

One question that remained was how N126K could provide increased resistance to RC-101 when it merely restored gp41 activity to that observed in the wild type. It is reasonable to assume that if N126K was in fact increasing fusion beyond what is seen in the wild-type virus, then the time in which gp41 is exposed to fusion inhibitors would decrease, and thus the kinetic window wherein fusion inhibitors exert their activity would be reduced as well. However, N126K only increased fusion when compared to Q66R alone, thus still decreasing the time that RC-101 could interact with gp41 while maintaining the resistance imparted by Q66R. This would explain the increase in RC-101 resistance as corresponding to a decrease in the time available for RC-101 to exert its activity.

A remaining question is why partial ENF resistance was achieved with the Q66R+N126K virus when N126K appears to only restore gp41 activity to that of the ENF-susceptible wild-type virus. Interestingly, Q66R has been identified in a patient receiving ENF treatment and may have been selected for by treatment [31]. In contrast, our experiments show that Q66R alone was not sufficient to provide any noticeable ENF resistance. This difference is possibly due to our studies utilizing the *env* derived from an R5 virus, rather than the more frequently studied X4 strains, which are known to display differences in entry rates, possibly due to utilization of separate coreceptors [32].

We have shown here that the same evolutionary path could achieve resistance to two distinctly different fusion inhibitors. Further, we have described the contribution of a secondary mutation responsible for the observed cross-resistance while exploring the mechanism by which resistance could be achieved. These findings were then applied to demonstrate how this mutation could provide improved resistance to other unique peptide entry inhibitors. Additionally, we show for the first time that RC-101 can inhibit the clinically significant enfuvirtide-resistant mutants V38A and V38+N126K. This insight provides us with direction in the continued development of fusion inhibitors and underscores the importance of compensatory HR2 mutations in drug-resistance.
